# The Effectiveness of Virtual Wards Compared to Inpatient Beds in Clinical Outcomes of Frail Older Patients With Chronic Conditions: A Systematic Review

**DOI:** 10.7759/cureus.97487

**Published:** 2025-11-22

**Authors:** Aretha Akinluyi, Oluseyi H Ogunfusika, Avril Brown

**Affiliations:** 1 Geriatrics, Leicestershire Partnership National Health Service (NHS) Trust, Leicester, GBR; 2 Urology, Kettering General Hospital, Kettering , GBR; 3 Community Health Sciences, Leicestershire Partnership National Health Service (NHS) Trust, Leicester, GBR

**Keywords:** chronic health conditions, digital monitoring, frailty, home telemonitoring, hospital at home, remote monitoring, telemedicine services, virtual ward

## Abstract

This systematic review evaluated the effectiveness of virtual wards (VWs) compared with traditional inpatient care for frail older adults with chronic conditions. VWs integrate multidisciplinary care and telehealth technologies to deliver hospital-level services at home, aiming to reduce unnecessary admissions and improve patient outcomes. A comprehensive search of five databases (Cochrane, CINAHL, PubMed, Science Direct, Google Scholar) identified 9,188 records. After removing duplicates and applying eligibility criteria, 52 full-text articles were screened, and six high-quality studies involving 2,325 participants met the inclusion criteria. Randomised controlled trials were appraised using the Cochrane Risk of Bias Tool (RoB 2), and quasi-experimental studies were assessed using the Joanna Briggs Institute (JBI) Critical Appraisal Checklist. Due to heterogeneity, a narrative synthesis was conducted.

Findings indicated that virtual wards supported by multidisciplinary teams and remote monitoring were associated with reduced hospital readmissions, improved quality of life, and high patient satisfaction. Some studies also demonstrated cost-effectiveness compared with inpatient care. However, variations in study design, population criteria, and outcome measures limited comparability, while mortality results were inconsistent. Barriers such as digital literacy, patient engagement, and access to technology also affected uptake and sustainability.

Overall, the evidence suggests that virtual wards can enhance outcomes for frail older adults and help relieve pressures on healthcare systems. Future research should adopt clearer definitions of frailty, standardised criteria, and consistent outcome measures to strengthen the evidence base. Larger, well-designed trials and long-term evaluations are needed to establish the clinical, social, and economic sustainability of virtual ward models. Sustained investment in digital infrastructure, workforce development, and patient engagement will be essential to support equitable and effective implementation across healthcare systems.

## Introduction and background

The number of older persons aged 75 years and older living in the UK has increased rapidly over the last decade. According to Pocock and Sharp, more than a third of the United Kingdom (UK) population lives up to at least the age of 85 years [1]. It is also estimated that 44 in every 1,000 older people are living with frailty [2]. The National Audit Office [3] also supports this, estimating that emergency admissions of older patients increased by 18% between 2010-11, and adding that older patients living with frailty now account for 62% of hospital beds. This implies that the prevalence of frailty increases with age [4].

Clegg et al. [5] define frailty as a state of vulnerability to adverse events from minor stressor events such as falls, leading to sudden health deterioration, prolonged hospitalisation, increased dependency, and even death. Unplanned early readmissions among older adults pose significant health, social, and financial challenges [6]. Consequently, readmission rates serve as commonly utilised benchmarks for assessing the quality of hospital care. Pocock et al. [7] suggest that almost two-thirds of patients aged over 85 die during a hospital admission, while Zisberg et al. [8] support that 40-50% of frail older people who eventually get discharged from the hospital go home with acute onset of disability or higher dependency in activities of daily living than pre-admission or return as failed discharges [9]. Ultimately, the prevalence of frailty increases with age and hospitalisation and is associated with functional decline, high mortality, and increased dependency.

In this context, in line with the National Health Service (NHS) Five Year Forward View [10], to address the overarching impact of frailty and to facilitate admissions avoidance, models such as frailty virtual wards (VWs), also known as hospital-at-home (HAH), have been developed [11]. VW began in the 2000s, particularly in the UK and the United States [12]; however, it gained renewed attention in the NHS during the initial phases of the COVID-19 pandemic. VWs are defined as healthcare models that are designed to provide hospital-level care to patients in their own homes through remote monitoring, multidisciplinary team support, and coordinated community-based interventions [12], with the aim to reduce unnecessary admissions, improve quality of life, and support health system capacity. This has the potential to reduce the increasing prevalence of frailty by decreasing hospitalisations, deconditioning, and promoting independence and a better quality of life. This review evaluates VW compared with inpatient beds for frail older adults with long-term conditions.

## Review

Study selection

**Figure 1:**
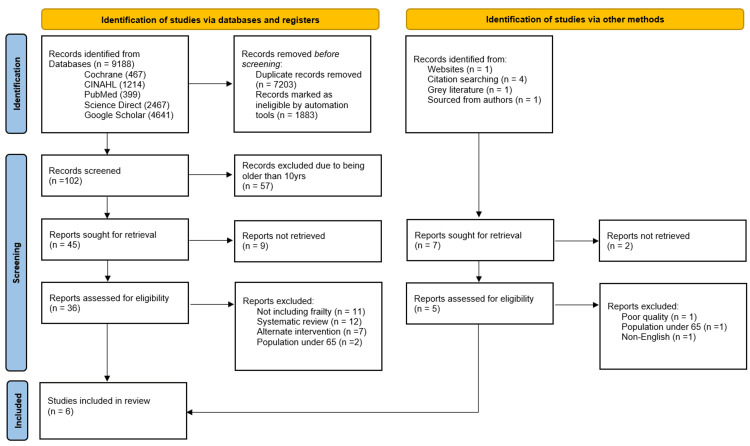
Study screening flow diagram per PRISMA 2020 guidelines PRISMA: Preferred Reporting Items for Systematic Reviews and Meta-analyses.

This review was conducted in accordance with the Preferred Reporting Items for Systematic Reviews and Meta-analyses (PRISMA), 2020 guidelines. An extensive search across PubMed, CINAHL, Cochrane Library, ScienceDirect, and Google Scholar yielded a substantial corpus of 9,188 records. The search incorporated a combination of keywords and medical terms: "virtual wards," " inpatient beds," "frail older adults," "long-term conditions," "clinical outcomes," "telehealth," and "remote monitoring (see Table 1). The search was limited to peer-reviewed articles published in English and subjected to rigorous title and abstract screening in line with predefined eligibility criteria, ultimately yielding six eligible papers of high quality, as detailed and rationalised in Figure 1.

**Table 1:** Search strategies

Database	Search Terms	Boolean Operators	Filters
PubMed (MEDLINE)	"Virtual wards" OR "remote monitoring" OR "hospital at home" OR "telehealth"	AND	Published in the last ten years, English, Peer-reviewed
	"Inpatient beds" OR "traditional care models" OR "usual care"		
	"Frail older adults" OR "elderly patients" OR "aged 65 and above."		
	"long-term conditions" OR "chronic conditions" OR "multimorbidity"		
	"Clinical outcomes" OR "hospital readmissions" OR "emergency department visits" OR "mortality rates" OR "patient satisfaction" OR "quality of life"		
CINAHL	"Virtual wards"	AND	Published in the last ten years, English, Peer-reviewed
	"Inpatient beds"		
	"Frail older adults"		
	"long-term conditions"		
	"Clinical outcomes"		
Cochrane Library	"Virtual wards"	AND	Published in the last ten years, Trials and Reviews
	" Inpatient beds"		
	"Frail older adults"		
	"long-term conditions"		
	"Clinical outcomes"		
Science Direct	"Virtual wards"	AND	Published in the last ten years, English, Peer-reviewed
	" Inpatient beds"		
	"Frail older adults"		
	"long-term conditions"		
	"Clinical outcomes"		
Google Scholar	"Virtual wards"	AND	Published in the last ten years, English, Peer-reviewed
	" Inpatient beds"		
	"Frail older adults"		
	"long-term conditions"		
	"Clinical outcomes"		

Inclusion Criteria

For the purpose of this study, VW were operationally defined as structured programmes delivering hospital-equivalent care in a patient’s home, supported by remote monitoring technologies and input from medical, nursing, and allied health professionals. Studies were coded as representing virtual ward interventions if they included: (1) clinician-led remote monitoring, (2) multidisciplinary care coordination, and (3) outcome measures related to hospital avoidance, readmission, or patient well-being. These criteria ensured consistency in identifying and analysing relevant evidence across the reviewed literature.

We included studies published within the last 10 years to reflect current practices and recent technological advancements. These studies focused on frail older patients aged 65 years and above with long-term conditions. The intervention of interest was the use of virtual wards, and we included only studies that compared virtual wards with traditional inpatient beds. Our outcomes of interest were hospital readmissions, emergency department visits, mortality rates, patient satisfaction, and quality of life. These were selected to provide a comprehensive understanding of the topic. We deemed only peer-reviewed studies suitable for inclusion to maintain high-quality standards and the credibility of the results.

Exclusion Criteria

Studies that did not report relevant clinical outcomes, such as hospital readmissions, emergency department visits, mortality rates, patient satisfaction, and quality of life, were excluded. Studies that did not focus on older adults aged 65 and above with multimorbidity and frailty were also excluded. Studies that did not involve the VW, HaH, or telehealth home-based care as the primary intervention were excluded. Also, studies that were not peer-reviewed, such as case reports, commentaries, editorials, and conference abstracts, were excluded. Studies that needed to provide more context on the healthcare system, especially those not comparable to the UK's NHS, were excluded. 

Quality assessment

Six studies were included in this review [13-18]. The methodological quality of the three Randomised Controlled Trials (RCTs) [13,14,16] was assessed using the Cochrane Risk of Bias Tool (RoB-2), in accordance with evidence-based medicine standards. This allowed classification into high-, unclear-, and low-risk of bias, which was performed independently by two researchers. The Quasi-experimental studies [15,17,18] were evaluated using the JBI critical appraisal checklist. The use of complementary appraisal tools was aimed at minimising the potential for selection, sampling, performance, and reporting bias (Tables 2, 3).

**Table 2:** Cochrane risk of bias for randomized controlled trials: judgment and justification

Criteria	Judgment	Reason/Justification
Random sequence generation	Low Risk High risk	Randomisation sequence not fully defined in Berglund et al., 2014 [13] whilst Shepperd et al., 2021 [14] and Olivari et al., 2018 [16] used computer-generated randomisation sequences to allocate participants to intervention or control groups, minimising selection bias.
Allocation concealment	Low Risk	Allocation concealment was achieved through sealed, opaque envelopes or secure web-based systems, ensuring that group allocation was unknown to participants before enrollment in all studies.
Blinding of participants	High Risk	Across all studies, participant Binding was not feasible due to the nature of the interventions (virtual wards/continuum at home vs. inpatient care), which could introduce performance bias.
Blinding of personnel	High Risk	Across all studies, binding healthcare providers was not possible because they were directly involved in delivering the interventions, which could introduce performance bias.
Blinding of outcome assessment	Low Risk	Outcome assessors were blinded to group allocation, reducing detection bias when evaluating primary and secondary outcomes in all studies.
Incomplete outcome data	Low Risk	Shepperd et al., 2021 [14] and Olivari et al., 2018 [16] reported low attrition rates. They used intention-to-treat analysis to handle missing data, minimising the impact of incomplete data on the study results.
Selective reporting	Low Risk	All three studies reported all pre-specified primary and secondary outcomes in their published protocols, ensuring transparency and reducing the risk of selective reporting.
Other biases	Low Risk	No other apparent biases were identified in the studies, and all followed standardised protocols for implementing and assessing the interventions, ensuring consistency and reliability in the findings.

**Table 3:** The revised JBI critical appraisal for the assessment of risk of bias for quasi-experimental studies

		Leung et al., 2015 [15]	deStampa et al., 2014 [17]	Low et al., 2015 [18]
Is it clear in the study what the “cause” and what the “effect” are (i.e., there is no confusion about which variable comes first)?		Yes	Yes	Yes
Bias related to selection and allocation				
Was there a control group?		Yes	Yes	No
Were participants included in any similar comparisons?		Yes	Yes	No
Bias related to the administration of the intervention/exposure				
Were the participants in any comparisons receiving similar treatment/care other than the exposure or intervention of interest?		Yes	Yes	No
Were there multiple outcome measurements, both pre- and post-intervention/exposure?		Yes	Yes	Yes
Were the outcomes of participants included in any comparisons measured in the same way?		Yes	Yes	Yes
Were outcomes measured in a reliable way?		Yes	Yes	Yes
Was the follow-up complete, and if not, were the differences between groups in terms of their follow-up adequately described and analysed?		Yes	Yes	Yes
Statistical Conclusion Validity				
Was an appropriate statistical analysis used?		Yes	Yes	Yes
Judgement		Include: ☒ Exclude: ☐	Include: ☒ Exclude: ☐	Include: ☒ Exclude: ☐

Characteristics of included studies

The studies included in this review span diverse geographical settings and research designs and explore the impact of virtual wards and home-based care models for frail older adults with chronic conditions. The first study, Berglund et al., was a randomized controlled trial conducted in Sweden involving 161 participants [13]. It investigated the effect of a multidisciplinary continuum-of-care intervention delivered in the homes of frail older adults compared to usual care. Participants were aged 65 years and above and required assistance with activities of daily living, with at least one chronic condition. The intervention involved coordinated care led by a multidisciplinary team. The primary outcome was life satisfaction measured at baseline and at three, six, and 12 months using the LiSat-11 scale, while secondary outcomes included illness, quality of life, functional ability, and medication use. The study found improved functional capacity and physical health among the intervention group at 12 months, though the differences in overall life satisfaction were not statistically significant. The second study by Shepperd et al. was a large, multicentre, randomized controlled trial conducted in the United Kingdom with 1,055 participants [14]. It evaluated a geriatrician-led hospital-at-home (HaH) service using comprehensive geriatric assessment (CGA) for older adults with frailty compared to standard inpatient admission. Participants were aged 65 years and older and had been referred for HaH care. The primary outcome was the proportion of participants living at home six months after randomization. Secondary outcomes included incidence of delirium, mortality, long-term residential care, cognitive impairment, activities of daily living, and quality of life. Findings indicated similar survival and functional outcomes between groups at 6 and 12 months, but fewer admissions to long-term care, greater patient satisfaction, and cost savings in the HaH group, supporting the feasibility of geriatrician-led virtual ward models. Thirdly, Leung et al. conducted a matched-control quasi-experimental pilot study in Hong Kong with 80 participants to examine the effectiveness of a virtual ward program that provided home visits and health monitoring for frail older adults at high risk of hospital readmission [15]. Participants were identified using a high-risk scoring tool (HARRPE >0.4). The study compared the virtual ward model with usual community nursing care. The primary outcome was a reduction in unplanned emergency readmissions, while secondary outcomes included emergency attendance and quality of life, measured using a locally validated quality-of-life questionnaire (mQOLC-E). Results demonstrated a greater reduction in unplanned readmissions and improved quality-of-life scores in the virtual ward group. Mortality was slightly higher in the intervention group, though not statistically significant, and was attributed to patient preference for receiving end-of-life care at home. Another study by Olivari et al. [16] was a randomized trial conducted across Italy and Greece involving 339 elderly patients who had been recently discharged from hospital after hospitalization for chronic heart failure. The study assessed the effectiveness of remote monitoring (RM) using wearable and telehealth devices compared with usual care. The intervention group received digital weight scales and wrist monitors that transmitted data daily to regional health centres for clinicians' review. The primary outcome was the 12-month incidence of hospital readmission for heart failure, while secondary outcomes included all-cause mortality and quality of life using the SF-36v2 scale. The results showed no significant differences in readmissions or mortality between the RM and control groups. However, participants in the RM group reported improved physical and cognitive functioning and better overall quality of life. The fifth study by de Stampa et al. was a pre-post quasi-experimental study with a control group conducted in France and included 428 participants [17]. It evaluated the COPA (Coordination des Personnes Âgées) program. This integrated home-based care model coordinates primary physicians and nurse case managers for frail older adults with complex health and social needs. The study examined the impact of COPA on hospitalizations compared with usual care. The primary outcome was any hospitalisation, planned or unplanned, while secondary outcomes included geriatric syndromes; cognitive function, continence, physical functioning, and mood, which were assessed using the RAI-HC scale. The study found a significant reduction in unplanned hospital admissions and improvements in functional and mental health parameters among the intervention group. The final study included in this systematic review, conducted by Low et al., was a pre-post quasi-experimental study conducted in Singapore involving 262 patients enrolled in a transitional home care (THC) program [18]. The intervention was led by a geriatrician supported by nurse case managers and aimed to reduce hospital admissions and emergency department attendance following discharge. Eligible participants were older adults with multimorbidity and subacute conditions requiring home-based follow-up. The primary outcomes were hospital admissions and emergency department attendances, with secondary outcomes including length of hospital stay, cost, and patient satisfaction. Findings demonstrated a 51.6% reduction in emergency readmissions at three months and a 52.8% reduction at six months. The average hospital stay decreased from 20.03 days to 12.05 days, resulting in estimated cost savings of approximately S$4.87 million. These results suggest that transitional home care and virtual ward models can provide significant cost savings and reduce acute hospital utilization. A table outlining the characteristics of included studies is shown in Table 1. 

**Table 4:** Characteristics of included studies

Section / Item	Berglund et al., 2014 [13]	Shepperd et al., 2021 [14]	Leung et al., 2015 [15]	Olivari et al., 2018 [16]	de Stampa et al., 2014 [17]	Low et al., 2015 [18]
Country / Setting	Sweden	United Kingdom	Hong Kong	Italy & Greece	France	Singapore
Study Design	Randomised controlled trial (RCT)	Randomised controlled trial (RCT)	Matched-control quasi-experimental pilot study	Randomised trial	Pre–post quasi-experimental study with control group	Pre–post quasi-experimental study
Sample Size	161 participants	1,055 participants	80 participants	339 participants	428 participants	262 participants
Participant Characteristics	Frail older adults with comorbidities	Frail older adults with complex health needs	Frail elderly post-discharge with functional disability	Elderly post-hospitalisation for chronic heart failure	Very frail elderly with complex health and social needs	Multimorbidity requiring subacute care
Inclusion / Exclusion Criteria	Inclusion: Adults ≥65 needing ADL assistance, ≥1 chronic condition, and frailty. Exclusion: Severe acute illness, dementia, palliative care.	Inclusion: Adults ≥65, English-speaking, referred to geriatrician-led HaH via CGA. Exclusion: Acute coronary syndrome, stroke, end-of-life care, unsafe home.	Inclusion: HARRPE score >0.4, older adults at high readmission risk, supported by carers. Exclusion: Physical or psychological inability to communicate.	Inclusion: Age >65, post-hospitalisation for heart failure. Exclusion: Severe comorbidity with life expectancy <12 months, inability to use telehealth, transplant waiting list, or trial enrolment.	Inclusion: Age ≥65, frail elderly with complex health and social needs. Exclusion: Planned institutionalisation or terminal illness.	Inclusion: Older patients with ≥3 comorbidities, subacute, and homebound requiring follow-up. Exclusion: No caregiver or independent in ADLs.
Intervention Description	Multidisciplinary continuum of care at home	Geriatrician-led admission avoidance hospital-at-home (HaH)	Virtual ward with home visits and monitoring	Remote monitoring using wearable devices and telehealth	COPA model (coordinated care for the elderly), geriatrician-led	Transitional home care with comprehensive needs assessment and follow-up
Comparator / Control Group	Usual care	Traditional inpatient admission	Usual care	Usual care	Usual care	No true control group
Primary Outcome(s)	Life satisfaction (LiSat-11) at baseline, 3, 6, 12 months	Living at home six months post-randomisation	Reduction in unplanned emergency readmissions	Combined 12-month incidence of HF readmission	Any hospitalisation (unplanned and planned)	Hospital admissions and ED attendances
Secondary Outcome(s)	Illness, QoL, functional ability, medication use, quality of care	Delirium, mortality, long-term care, cognition, ADLs, QoL, length of stay, readmission	ED attendance, QoL	All-cause mortality, QoL	Physical, cognitive, communicative, mood, and functional health parameters	Hospital length of stay, specialist visits, patient and caregiver satisfaction
Limitation(s)	Intervention began before baseline; LiSat-11 has not been validated for older adults.	Established HaH service limited generalisability beyond mature sites	Small sample, time constraints, no cost data	Recruitment interruption in Greece reduced statistical power	Non-randomised design; one-year follow-up only	Self-controlled design; incomplete cost data

Data synthesis and analysis

Data from the included studies were extracted using a standardised data extraction form. The extracted data included study characteristics (author, year, country, study design), intervention characteristics (type of virtual ward, duration), comparison group details, and reported outcome. A mixed-methods approach was employed for data analysis. Quantitative data were synthesised by providing pooled effect estimates with 95% confidence intervals (CIs). Qualitative data were analysed thematically to identify common themes and patterns related to patient and caregiver experiences, technological challenges, and equity issues.

**Table 5:** Data extraction table

Author	Title	Aim	Method	Sample size	Sampling Technique	Findings
Berglund et al., 2014 [13]	Effects of a continuum of care intervention on frail older persons’ life satisfaction: a randomised controlled study	To analyse the effects of a comprehensive continuum of care (intervention group) on frail older persons’ life satisfaction, as compared to those receiving usual care (control group).	RCT	161	Random allocation	The study found that at the 12-month follow-up, a higher percentage of participants in the intervention group reported satisfaction, with a statistically significant difference observed for physical health (OR 2.57, CI 1.19–5.55). However, at the 6-month follow-up, there was a significant difference in favour of the control group for psychological health (OR 0.45, CI 0.24–0.86). Between the 6- and 12-month follow-ups, the intervention group showed greater improvement or maintenance of satisfaction than the control group. Statistically significant odds ratios were found for functional capacity, psychological health, and financial situation.
Shepperd et al., 2021 [14]	Is Comprehensive Geriatric Assessment Admission Avoidance Hospital at Home an Alternative to Hospital Admission for Older Persons?	To assess the clinical effectiveness of admission avoidance hospital at home (HAH) with CGA for older persons.	Randomised Control Trial	1,055	Random allocation	The study found no significant difference in living at home at 6 months (relative risk [RR], 1.05 [95% CI, 0.95 to 1.15]; P = 0.36) or 12 months (RR, 0.99 [CI, 0.89 to 1.10]; P = 0.80), nor in the risk for death or cognitive impairment at 6 months. However, there was a notable decrease in long-term residential care in the HAH group at 6 months (RR, 0.58 [CI, 0.45 to 0.76]; P < 0.001) and 12 months (RR, 0.61 [CI, 0.46 to 0.82]; P < 0.001). Additionally, the HAH group showed an increased risk of readmission or hospital transfer at 1 month but not at 6 months (RR, 0.95 [CI, 0.86 to 1.06]; P = 0.40).
Leung et al., 2015 [15]	The effect of a virtual ward program on emergency services utilisation and quality of life in frail elderly patients after discharge: a pilot study	To examine the impacts of the virtual ward service on changes in the patients’ emergency attendance and medical readmissions, and their quality of life (QOL)	quasi-experimental study	80	Matched case-control sampling	The group that received intervention demonstrated a notably greater decrease in the number of unplanned emergency hospital admissions compared to the control group (-1.41±1.23 versus -0.77±1.31; P=0.049). However, there were no significant differences in the length of stay and the number of emergency attendances between the two groups (-1.51±1.25 versus -1.08±1.48; P=0.29). In terms of quality of life (QOL), patients who received the virtual ward service reported higher average scores in the overall score changes and scores in all six dimensions of mQOLC-E.
Olivari et al., 2018 [16]	The effectiveness of remote monitoring of elderly patients after hospitalisation for heart failure: The renewing health European project?	to explore the effectiveness of remote monitoring in elderly patients with heart failure	Randomised control trial	339	Random allocation	No difference in all-cause mortality in the intervention group vs usual care (24.0% vs 21.8%, p = 0.097) or the rate of patients with at least one hospital readmission for HF (34.5% vs 39.1%, p = 0.48). However, a significantly better outcome in QoL, physical and mental status was reported as compared with the UC group.
De Stampa et al., 2014 [17]	Impact on hospital admissions of an integrated primary care model for very frail elderly patients	to assess the impact of this model on hospital admissions and health parameters among community-dwelling very frail elderly patients	quasi-experimental study	428	Pre-post test with control group	The intervention group had a lower risk of unplanned hospital admission (OR = 0.39; 95% CI = 0.16–0.98). Additionally, there was a non-significant decrease in total hospital admissions in the intervention group (OR = 0.75, 95% CI = 0.36–1.58). Furthermore, lower risks of depression was noted in the intervention group compared to the control group at 12 months (OR = 0.42, 95% CI = 0.20–0.90), with similar results for dyspnoea (OR = 0.26, 95% CI = 0.09–0.77).
Low et al., 2015 [18]	Effectiveness of a transitional home care program in reducing acute hospital utilisation: a quasi-experimental study	to evaluate the effectiveness of such a program that is operated by the Singapore General Hospital (SGH) in reducing acute hospital readmission and ED visits	quasi-experimental study	262	pre-post design without matched-control	a 51.6% reduction in ED readmission at three months (p < 0.001) and 52.8% at six months. Additionally, the hospital stays per patient reduced significantly from 20.03 days in three months to 12.05 days at six months. 5,787 bed days saved with an associated cost savings of $4.87 million and 301 fewer ED attendances with associated cost savings of $65,016 reported

Discussion

Six high-quality studies, encompassing 2,325 participants, met the inclusion and quality criteria for this review. Collectively, these studies demonstrate that virtual wards (VWs) are associated with reduced hospital readmissions, improved patient-reported outcomes, and higher satisfaction among frail older adults with chronic conditions when compared with conventional inpatient care. The evidence suggests that VWs, by integrating remote monitoring, multidisciplinary team (MDT) input, and patient-centred home-based care, offer a viable and often preferable alternative to traditional hospitalization.

Overall effectiveness

Across studies, VW interventions consistently achieved lower readmission rates and improved secondary outcomes, including medication adherence and functional independence [13-15]. Early detection of deterioration, medication reconciliation, and polypharmacy management are key mechanisms in mitigating preventable readmissions [15,16]. They emphasized the importance of timely home visits and team coordination, while [18] found that adherence to monitoring protocols was critical to success. Two studies [14,18] reported economic benefits of VW implementation through shorter lengths of stay and fewer emergency department visits. Although some studies described resource-intensive initiation [16,17], long-term savings through reduced inpatient utilization were evident. Patient-centred outcomes were also favourable. All studies except [18] demonstrated improved quality of life (QoL) and satisfaction [14,15,17]. Mortality rates were similar to usual care; [15] reported a non-significant increase in mortality (p = 0.27) attributed to patients in advanced disease stages who opted for home-based palliation.

Theme 1: Patient Satisfaction

Four studies [14,15,17,18] reported high patient satisfaction, reflecting confidence in VW Care's patient-centred approach. Satisfaction was linked to continuity of care, comfort of the home environment, and consistent MDT support. A recent review [19] reported increased satisfaction among hospital-at-home patients and staff, as well as improved quality of care, reflecting the outcomes evaluation [20]. Most VW interventions appear to improve both domains by promoting personalized interaction and relational continuity. Ease of use of remote monitoring devices also contributed to autonomy and dignity [21,22]. Only [16] used continuous wearable devices, indicating an underexplored technological dimension. The inclusion of physiotherapists and occupational therapists, as shown in [13], enabled real-time rehabilitation planning and home-based functional assessment, aligning with [23], which found that satisfaction declines when expectations are unmet.

Theme 2: Cost-Effectiveness

Cost-effectiveness emerged as a significant advantage of VWs. A reduction in mean hospital stay from 20.03 to 12.05 days, saving 5,787 bed days and approximately $4.87 million, was reported by [18]; Emergency department visits decreased by 301, saving $65,016. Similarly, [14] found reduced healthcare utilization without compromising outcomes. [22] reported that tele-rehabilitation participants had significantly fewer readmissions (0.49 vs 1.17 per patient, P = 0.041) and lower costs (€3,461 vs €4,576 per patient). [24] showed that hospital-at-home care was 38% cheaper than inpatient care (p < 0.001), although cost variation between interventions remains high. While [16] and [17] noted the resource intensity of establishing VW programs, the overall cost-benefit was favourable once reduced admissions were accounted for.

Theme 3: Quality of Life

Most studies demonstrated significant improvements in QoL among VW participants [13-16]. Gains were measured using validated tools such as the SF-36v2 and LiSat-11 scales and attributed to continuous, holistic, and patient-empowering care. [16] observed reduced all-cause mortality among heart failure patients, highlighting potential life-preserving effects of remote monitoring. Using the COPA model, [17] demonstrated improvements across mental and functional domains, indicating comprehensive health benefits.

Theme 4: Readmission Rates

All included studies reported reductions in hospital readmissions following VW implementation [14-16,18]. A previous study by [25] documented a 2-6% reduction in readmissions with pharmacist-led BOOST (Better Outcomes for Older adults through Safe Transitions) discharge coordination, findings supported by [26], which cited a 15% decrease in 30-day readmissions among patients with follow-up discharge care. [27] observed a 40% reduction in bed days during the COVID-19 VW deployment. Conversely, [28] reported no significant difference between community rehabilitation and inpatient rehabilitation, suggesting that program intensity and patient selection are crucial.

Collectively, the evidence indicates that VWs improve clinical outcomes, enhance satisfaction, and optimize healthcare resources among frail older adults with chronic diseases. The multidisciplinary, proactive, and home-based model contrasts with the episodic nature of hospital care and addresses key contributors to avoidable readmissions.

Strengths and limitations

This review synthesised a diverse range of studies evaluating interventions for frail older adults with long-term conditions. The inclusion of both randomised controlled trials (RCTs) and quasi-experimental designs strengthened the evidence base and enhanced comparability. Integrating quantitative and qualitative findings provided not only measurable outcome data but also valuable contextual understanding. However, several limitations should be acknowledged. A clear and consistent definition of the target population, including how frailty was measured or indexed, was not always evident across studies. Variations in the application of frailty indices may have influenced comparability between studies and the interpretation of outcomes.

In addition, there are potential confounding risks related to study selection. More clinically complex patients may have been retained in inpatient settings, while virtual ward interventions may have been preferentially offered to lower-risk individuals. Such selection bias warrants cautious interpretation of reported outcomes and limits the ability to draw firm causal inferences about intervention effectiveness.

A further key limitation was the inability to perform a meta-analysis due to significant heterogeneity among the included studies. Substantial variation in study design, population characteristics, outcome measures, and the nature of virtual ward interventions restricted opportunities for statistical pooling. Combining data from such diverse interventions and populations could potentially lead to misleading pooled estimates. Consequently, findings were narratively synthesised to capture the range and complexity of approaches implemented across healthcare settings. While this approach provided a broader understanding of virtual ward models, it may also limit the generalisability of the conclusions drawn.

The absence of randomisation in one quasi-experimental study may have introduced additional bias, and the predominance of pilot-stage interventions further reduced external validity. Additionally, restricting inclusion to English-language studies may have introduced language bias, as potentially relevant evidence published in other languages was excluded. This limitation may affect the completeness and global representativeness of the findings. Despite these limitations, this review offers valuable insights into the effectiveness of virtual ward interventions and underscores the need for more rigorous, standardised, and diverse research to strengthen future evidence.

Recommendations for future research

Future research should address the methodological limitations identified in this review by adopting clearer and more consistent definitions of frailty, alongside standardised population criteria and frailty indexes. Studies should give greater attention to minimising selection bias and confounding factors to enable more accurate comparisons between inpatient and virtual ward populations. The use of consistent outcome measures and intervention frameworks would also reduce heterogeneity and improve the feasibility of meta-analyses.

To strengthen the evidence base and inform evidence-based policy, larger randomised controlled trials and well-designed quasi-experimental studies are needed. Long-term evaluations should examine the sustainability of virtual ward benefits across clinical, social, and economic outcomes. Future studies should also investigate how digital inclusion, patient engagement, and carer support influence adherence and effectiveness, as well as undertake comparative analyses of cost-effectiveness and system-level impacts across different contexts.

## Conclusions

This review highlights the potential of virtual ward models as effective alternatives to inpatient care for frail older adults with long-term conditions. Evidence suggests they can reduce hospital readmissions, enhance continuity, and improve quality of life when well integrated into community and primary care pathways. Sustained investment in digital infrastructure, workforce development, and service redesign is needed to ensure safe and equitable implementation. Policy support should prioritise training, multidisciplinary collaboration, and patient and caregiver engagement to maintain person-centred care.

Future research should adopt clearer definitions of frailty, standardised criteria, and consistent outcome measures. Larger, well-designed trials and long-term evaluations are required to assess sustainability, cost-effectiveness, and system-level impact. With coordinated policy and rigorous evaluation, virtual wards can contribute meaningfully to more efficient and responsive health and social care.
